# Research priorities in emergency general surgery (EGS): a modified Delphi approach

**DOI:** 10.1186/s13017-022-00432-0

**Published:** 2022-06-16

**Authors:** Elizabeth Mary Vaughan, Robert Pearson, Jared Mark Wohlgemut, Stephen Richard Knight, Harry Spiers, Dimitrios Damaskos, Julie Cornish, Chetan Parmar, Kamal Mahawar, Susan Moug, Gian Luca Baiocchi, Fausto Catena, Gillian Tierney, Michael Samuel James Wilson, the Scottish Surgical Research Group, the World Society of Emergency Surgery and the Association of Surgeons of Great Britain & Ireland

**Affiliations:** 1 Department of General Surgery, Bradford Royal Infirmary, Bradford, BD9 6RJ UK; 2 Department of General Surgery, Elizabeth University Hospital, Glasgow, G51 4TF Queen UK; 3 Blizard Institute of Cell and Molecular Science: Barts and The London School of Medicine and Dentistry Blizard Institute, Whitechapel, London, E1 2AT UK; 4 The University of Edinburgh Usher Institute of Population Health Sciences and Informatics, University of Edinburgh, Edinburgh, EH16 4UX UK; 5 Department of General Surgery, Addenbrooke’s Hospital, Cambridge, CB2 0QQ UK; 6 Department of General Surgery, Royal Infirmary Edinburgh, Edinburgh, EH16 4SA UK; 7 Department of General Surgery, University Hospital Wales, Cardiff, CF14 4XW UK; 8 Department of General Surgery, The Whittington Hospital NHS Trust, London, N19 5NF UK; 9 Department of General Surgery, Sunderland Royal Hospital, Sunderland, SR4 7TP UK; 10 Department of General Surgery, Royal Alexandra Hospital, Paisley, PA2 9PJ UK; 11 Department of Clinical and Experimental Sciences, University of Brescia, ASST Spedali Civili, Brescia, Italy; 12 Department of Emergency and Trauma Surgery, University of Parma, Parma, Italy; 13 Department of General Surgery, Derby City General Hospital, Derby, DE22 3NE UK; 14 Department of General Surgery, Forth Valley Royal Hospital, Larbert, FK5 4WR UK

**Keywords:** Emergency, General, Surgery, Research, Delphi

## Abstract

**Background:**

Emergency general surgery (EGS) patients account for more than one-third of admissions to hospitals in the National Health Service (NHS) in England. The associated mortality of these patients has been quoted as approximately eight times higher than that of elective surgical admissions. This study used a modified Delphi approach to identify research priorities in EGS. The aim was to establish a research agenda using a formal consensus-based approach in an effort to identify questions relevant to EGS that could ultimately guide research to improve outcomes for this cohort.

**Methods:**

Three rounds were conducted using an electronic questionnaire and involved health care professionals, research personnel, patients and their relatives. In the first round, stakeholders were invited to submit clinical research questions that they felt were priorities for future research. In rounds two and three, participants were asked to score individual questions in order of priority using a 5-point Likert scale. Between rounds, an expert panel analysed results before forwarding questions to subsequent rounds.

**Results:**

Ninety-two EGS research questions were proposed in Phase 1. Following the first round of prioritisation, forty-seven questions progressed to the final phase. A final list of seventeen research questions were identified from the final round of prioritisation, categorised as condition-specific questions of high interest within general EGS, emergency colorectal surgery, non-technical and health services research. A broad range of research questions were identified including questions on peri-operative strategies, EGS outcomes in older patients, as well as non-technical and technical influences on EGS outcomes.

**Conclusions:**

Our study provides a consensus delivered framework that should determine the research agenda for future EGS projects. It may also assist setting priorities for research funding and multi-centre collaborative strategies within the academic clinical interest of EGS.

## Background

Patients requiring emergency general surgery (EGS) are known to have a high risk of death and complications [1–3] with estimates suggesting they account for 50% of all surgical mortality [3]. The provision of EGS service is a global public health issue, which has made it an important area of research [4, 5]. In the UK, the National Emergency Laparotomy Audit (NELA) projects have shown the benefits of research on quality improvement for EGS patients [6, 7].

With the emergence of EGS as an important area of clinical interest in need of service reconfiguration, there is growing momentum to address the challenges involved in the delivery of patient focused, safe and proficient care and to improve patient outcomes [8]. Identifying patient-centred research priorities has the potential to deliver clinically relevant questions that could guide funding and management strategies and prioritise resource allocation thereby strengthening standards of care provided within this speciality [9].

A modified Delphi process can be used to establish a consensus opinion on research priorities from a panel of experts within that field [10]. Using a participative approach and structured prioritisation methods, stakeholders can identify research that they believe will have the most meaningful impact on EGS care.

The aim of this research is to produce a peer-reviewed list of research priorities for EGS. The study was undertaken by members of the Scottish Surgical Research Group (SSRG) with organisational support provided by the World Society of Emergency Surgery (WSES) and Association of Surgeons of Great Britain and Ireland (ASGBI).

## Methods

**Fig. 1:**
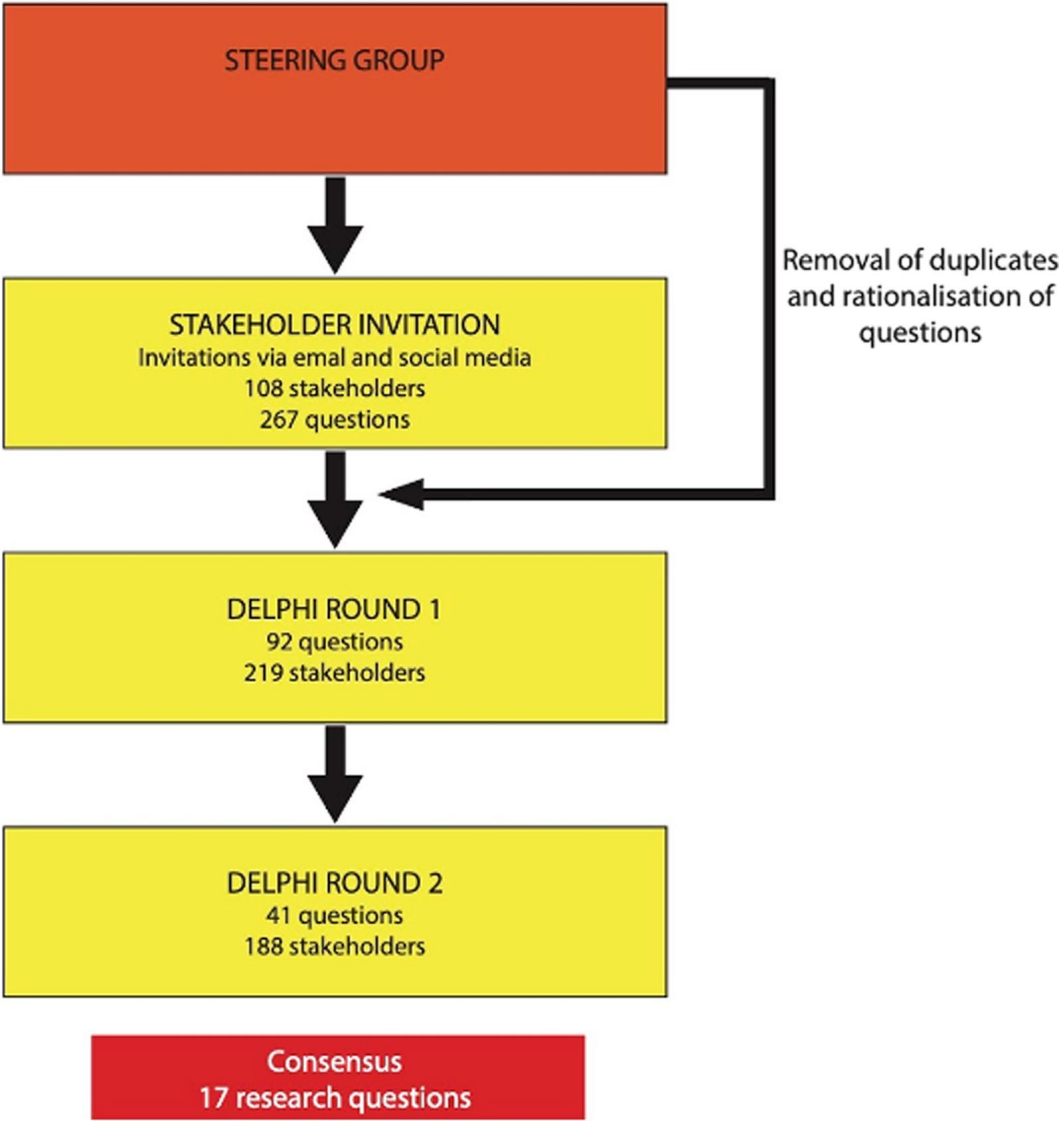
Summary of prioritisation process

A modified Delphi technique was used to identify research priorities in EGS and build consensus across a group of stakeholders (Fig. 1). The Delphi method is an a priori, structured communication technique in which a group of experts reach a structured consensus on a topic through a number of rounds of questions with controlled feedback [11, 12]. In order to be General Data Protection Regulation (GDPR) compliant, formal consent was gained to use responses. Respondents were also given the opportunity to withdraw consent. Respondents were allocated an anonymous ID, and identifiable data were not publicised. Research ethical approval was not required for this study according to National Research Ethics Service guidance [11].

### Stakeholder identification

A three-phased modified Delphi process was undertaken which included two phases of prioritisation by stakeholders using established methodology from other Delphi processes [12, 13].

The steering committee for this research consisted of five general surgery specialty trainees (EV, RP, JW, SK and HS) and nine consultant general surgeons (MW, DD, JC, SM, KM, CP, FC, GLB and GT), all of whom have an interest in EGS. The role of the steering committee was to ensure relevance of the submitted questions from both a clinical and patient perspective and to provide consensus agreement on amendments to submitted questions and cut-off points following prioritisation in Phases II and III.

### Phase I

Stakeholders were invited to submit research questions relevant to the field of EGS using an electronic questionnaire (https://www.surveymonkey.com). Invitation emails were sent to the ASGBI and WSES membership. Twitter(_R)_ (Twitter Inc., San Francisco, California) was also used to broaden the awareness of the Delphi process amongst interested stakeholders using a dedicated study account (@EmSurg_Delphi). There was no limit to the number of questions that an individual could submit. Questions were encouraged from doctors, nurses, allied health professionals as well as from patients who have undergone an EGS procedure or were admitted as an emergency under the care of a general surgeon. Phase I was open for 6 weeks.

Following submission of questions, a subcommittee categorised all questions into topics. These were EGS, emergency upper gastrointestinal surgery, emergency colorectal surgery, non-technical questions and health service questions. Duplicate questions were deleted. Questions with a similar theme were modified by consensus agreement, and care was taken not to alter the meaning of the questions.

### Phase II

ASGBI and WSES members were invited by email containing a link to an online survey to prioritise the consensus agreed list of questions from Phase I. Twitter was again used to broaden the awareness of the study among stakeholders. Using a Likert scale, stakeholders ranked questions from 1 (lowest research priority) to 5 (highest research priority). The survey remained open for 6 weeks with 1 email reminder sent to ASGBI and WSES members. The results were reviewed by the subcommittee.

Questions that were scored as 4 or 5 by ≥ 55% stakeholders were progressed to the final round of prioritisation. A 55% cut-off was chosen, without sight of the questions, as there was a clear gap in scores for questions below this percentage. It also yielded a number of remaining questions regarded as manageable for use in Phase II.

### Phase III

A final round of prioritisation was undertaken on the consensus agreed list of questions at the end of Phase II using the same methodology as before. A higher cut-off of 65% of questions being scored as 4 or 5 was agreed, again without sight of the questions for inclusion in the final list of prioritised questions. This phase stayed open for 6 weeks.

## Results

In total, 108 stakeholders submitted 267 individual research questions in Round I. Following analysis and categorisation by the steering subcommittee, 92 questions were forwarded for inclusion in the first phase of prioritisation (see Appendix—Table 2). The composition of the initial stakeholders included consultant surgeons (*n* = 70), registrar/fellow/specialty doctor (*n* = 21), physicians (*n* = 11), patients (*n* = 3), Senior House Officers (*n* = 2), one medical student, and one pharmacist.

In the first phase of prioritisation, 92 questions were prioritised by 219 stakeholders (Appendix—Table 1). These included 196 EGS health care professionals, 11 patients and 12 others (including public and relatives). In the second phase of prioritisation, 41 questions from Phase I were ranked by 188 stakeholders (Appendix—Table 3). Following review by the steering committee, a final list of 17 prioritised research questions was agreed.

## Discussion

Over the last decade, significant changes in the organisation, management and delivery of EGS services in units across the UK have resulted in improved service provision. A growing body of consultant surgeons with a special interest in EGS, combined with structural changes within these departments, have enabled the tailoring of strategic developments geared towards improving care for patients requiring EGS services. Many of these seismic shifts have been research driven. Studies reporting higher mortality rates with emergency laparotomies compared to elective cases, and others demonstrating wide variation in outcomes between trusts have highlighted the need for further research aimed at improving standards of care for emergency cases [1, 14, 15].

The National Confidential Enquiry into Patient Outcomes and Deaths (NECPOD) [16] and the National Emergency Laparotomy Audit (NELA) [6] were two studies designed to collect organised and comparative data on emergency service provision across the UK in an attempt to improve the quality of care for patients undergoing emergency surgery. The enhanced perioperative care for high-risk patients trial (EPOCH) [17] and the Emergency Laparotomy Collaborative (ELC) projects also focussed on areas in which patient outcomes could be improved. With approximately 25,000 patients undergoing emergency abdominal surgery annually in NHS hospitals with 30-day mortality rates of 9.6% [7], national clinical projects like these are essential. The Emergency Laparoscopic and Laparotomy Scottish Audit (ELLSA) aimed to capture an even more comprehensive EGS cohort than NELA by widening the inclusion of contributing sites and including laparoscopic procedures [18]. To our knowledge, this study is the first Delphi undertaken in the field of EGS and is intended to guide this much needed research and stimulate health care quality improvement.

**Fig. 2:**
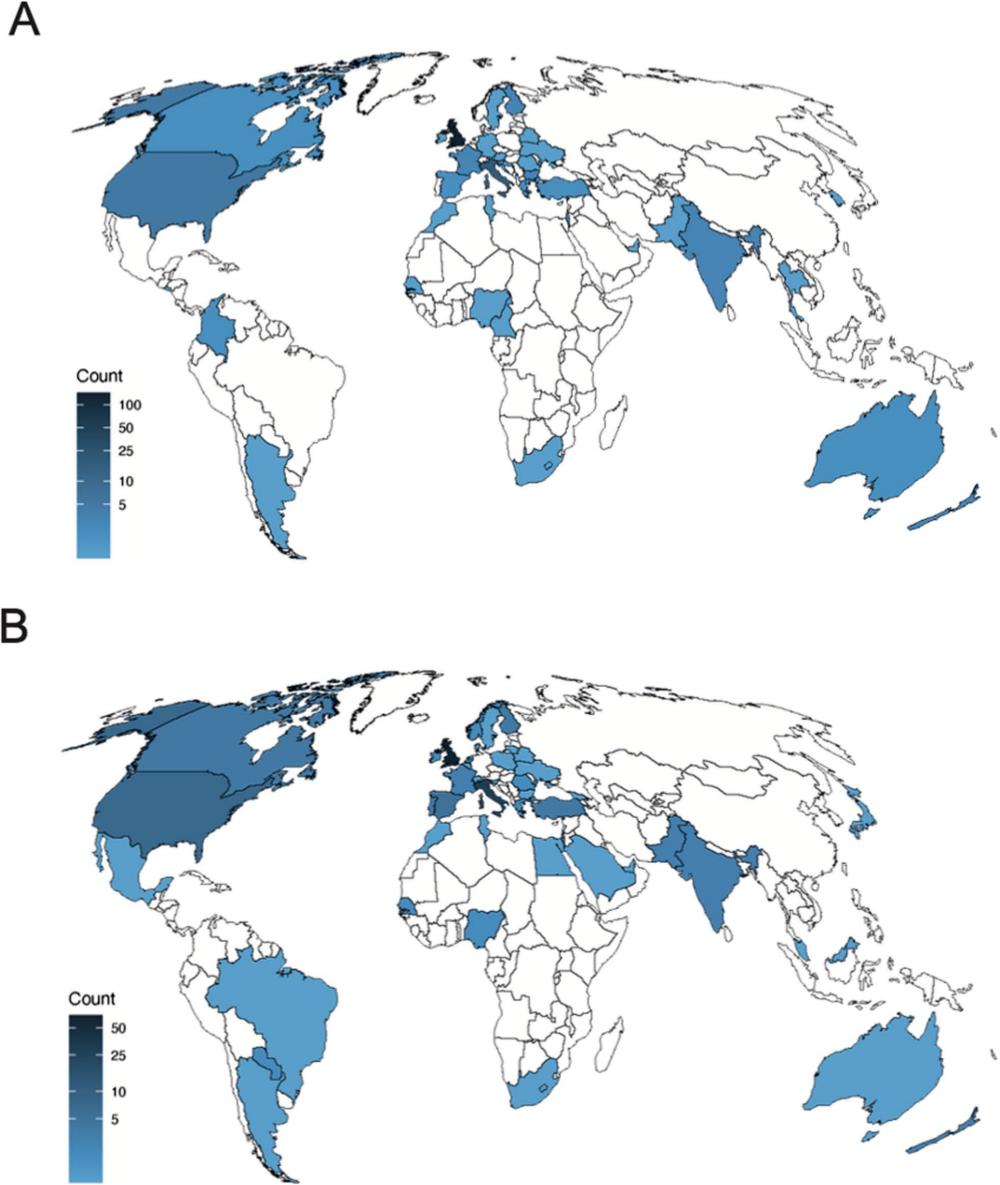
Distribution of respondents prioritising research questions in **A** the first and **B** second phase of prioritisation

Our modified Delphi process has produced a list of 17 high-priority research questions in the field of EGS. We adopted a non-biased approach of inviting members of two established surgical societies (ASGBI and WSES), but also publicised on Twitter in order to ensure that members of the public and patients were able to participate. Figure 2 demonstrates heat maps of the distribution of respondents prioritising research questions in Phase II (A) and III (B), respectively. The input of the latter two groups was a valued addition, with the focus of many research areas identified relating to patient experience and patient-reported outcomes.

There are no defined criteria on setting cut-off consensus levels in Delphi studies [19]. Consensus levels are defined as a percentage higher than the average percentage of majority opinion [20], and many researchers have used different levels of agreement to achieve consensus. As the aim of our study was not to achieve a pre-determined consensus level, each phase of our Delphi was terminated based on subjective analysis of the number of questions remaining after each round. Following the first round of prioritisation, in the case of a 55% majority there was consensus on 41 questions, a 60% majority resulted in 23 questions and at 70% concordance 7 questions remained. To produce a manageable number of relevant questions, a majority view within the steering group chose a level of agreement of 55% for the first round of prioritisation. We chose a more strict criteria of 65% for the final list of questions, again to produce a manageable number of questions with the highest priority.

There were a number of questions that did not make the final list of prioritised questions. A ranked list of all questions is included in the Appendix (Table 3).

The Emergency Laparotomy and Frailty (ELF) study highlighted that 20% of patients undergoing emergent laparotomy in the UK are frail, presenting greater risk of post-operative morbidity and mortality, independent of age [21]. In the fourth patient report of NELA [7], researchers identified that despite evidence of improved outcomes with comprehensive geriatric assessment methodology [22], there was no improvement in the proportion of patients over the age of 70 benefiting from geriatric specialist input. This is reflected in our study. From our list of prioritised questions, a recurrent theme was consideration on focusing future research on the management of older adult and frail patients undergoing EGS.

Our final list of prioritised questions also included a significant emphasis on optimisation of EGS services and training. Further studies are also required to develop a greater understanding of optimisation of EGS patients peri-operatively, and research into technical considerations in emergency colorectal surgery is required to guide potential improvements in survival outcomes. The study’s results are particularly relevant in the current setting.

One major limitation we anticipated was that of survey fatigue—the tendency to not fully complete a survey when faced with several pages of questions, or reluctance to participate at all. To mitigate this, we designed the surveys with categories in reverse order between surveys.

Another limitation was the lack of patient input into this project, which risks avoiding the research areas which are of interest to patients. The intention of the study group was to hold a patient focus group at the end of Phase I. However, this was not possible as the timing coincided with the COVID-19 pandemic and the first lockdown in the UK. It was therefore decided to abandon this aspect of the study in the interest of the safety of our patients and to focus on gathering the views of members within the EGS multidisciplinary care team. Though views of EGS patients and patients’ families were still sought, they did not yield many responses. Health charities and patient support groups are often keen participants in this type of research. However, there are relatively few EGS groups compared to conditions such as Crohn’s disease and colitis [23], or bowel cancer [24], highlighting that the EGS patient group is overlooked. There is clear scope to address this limitation of our study in the future.

A final limitation of EGS research to date is the overemphasis on mortality and morbidity as outcomes, which comprise a valuable future project.

We have used this modified Delphi method to survey multiple stakeholder groups including patients, health care providers and multidisciplinary team members involved in all aspects of EGS care provision. We believe that this is an important body of work that demonstrates consensus across a broad and diverse group of stakeholders. The findings of this study can be used to guide future research studies and research funding in the EGS community.

## Conclusions

Seventeen high-priority research questions relevant to EGS have been identified by a consensus of EGS stakeholders by a modified Delphi process. This work should be used to focus future EGS research and funding and to encourage high-quality patient-centred multi-centre, international studies.

## Data Availability

Not relevant.

## Appendix: Tables

See Tables 1, 2 and 3.

**Table 1:** Questions put forward for the first round of prioritisation

Question number	Question	High-priority ranking (4 or 5) (*n*)	Low-priority ranking (1 or 2) (*n*)
*General EGS*			
3	What is the impact of emergency laparotomy on quality of life in frail patients?	135	10
24	Which pre- or post-operative interventions improve clinical outcomes in patients with frailty after emergency surgery?	130	4
25	What are the best predictors of adverse outcomes and mortality in older adults presenting with emergency general surgery pathology?	124	10
16	How can training be improved to meet emergency general surgery demand, including open surgery?	119	13
4	What is the value of enhanced recovery after surgery (ERAS) programmes for EGS patients, compared to standard management?	114	12
26	What is the optimal method of assessing fitness for surgery in the emergency setting?	111	19
13	What are the absolute risks of anastomotic leak following emergency small bowel/large bowel anastomosis, and the relative importance of risk factors?	111	18
15	How can we optimise the rate of reversal of Hartmann’s when performed as an emergency and what are the predictive factors in those who are subsequently reversed?	108	15
19	How does small bites closure compare to traditional mass closure in the emergency setting (wound infection, fascial dehiscence and incisional hernia rates)?	101	25
17	Should patients have extended venous thromboembolism (VTE) prophylaxis after an emergency laparotomy with or without resection?	100	23
39	Is there a link between duration of emergency laparotomy (knife to skin to wound closure) and clinical outcomes? Is there a cut-off where all surgeons should perform damage control surgery in the emergency setting?	95	21
41	How can technology be incorporated into patient follow-up to aid early detection of post-operative complications?	95	25
46	Are outcomes after emergency surgery improved with the laparoscopic approach as compared to the open approach?	95	21
23	Does a laparoscopic approach for adhesional small bowel obstruction reduce the risk of recurrence, and how do we identify those who would benefit most from laparoscopic approach?	93	28
9	Is non-operative management inferior to the surgical management of uncomplicated appendicitis?	91	31
18	How does negative pressure wound therapy affect clinical outcomes after 90 emergency surgery?	28	
7	Which cohort of patients can be safely managed using an ambulatory appendicitis pathway or day-case appendicectomy for acute appendicitis?	87	33
22	What is the best diagnostic approach to acute small bowel obstruction, and what is the role of Gastrografin in diagnosis and/or treatment?	87	34
20	Is there value in routine computer tomography (CT) imaging in patients presenting with acute abdominal pain?	86	29
14	What is the role of the gut microbiome in anastomotic leakage?	86	31
34	Non-specific abdominal pain—how do you define, manage and reduce readmissions?	85	40
31	Does early parenteral nutrition in small bowel obstruction improve clinical outcomes?	82	27
1	What more can be done to help patients with the psychological sequelae of emergency surgery, and which patient-reported outcome measures are most useful?	82	29
11	Should we implement a UK wide acute CT service for right iliac fossa pain to reduce the negative appendicectomy rate?	81	35
5	Which cohort of EGS patients derive the greatest benefit from post-operative chest physio?	81	31
35	Does the early administration of inotropes during the resuscitation of patients with septic shock result in improved clinical outcomes?	80	29
37	What is the optimal method of antibiotic stewardship and rationalisation in emergency general surgery?	80	42
10	Is there a difference in clinical outcomes between patients with image-proven appendicitis and conventional (clinically guided) appendicitis?	79	40
45	Is the use of mesh safe for the repair of a strangulated hernia where there has been small bowel contamination via enterotomy?	79	41
29	Which patients are most likely to need parenteral nutrition following emergency surgery?	75	43
33	What are the indications for diagnostic laparoscopy in women of reproductive age presenting acutely with lower abdominal pain?	75	49
6	Which diagnostic tool is the most sensitive and specific for acute appendicitis?	73	46
30	What is the optimal resuscitation fluid for patients undergoing emergency laparotomy?	70	47
21	Does point-of-care ultrasound (POCUS) improve clinical outcomes in emergency surgery patients?	70	41
27	Is CT-diagnosed sarcopenia useful as a prognostic indicator of poor outcomes in emergency surgical patients?	66	49
32	What is the value of nutritional supplements (e.g. Fortisip) to EGS patients?	63	35
43	Is there a role for a symptomatic hernia ‘hot list’ and would it improve clinical outcomes in this cohort of patients?	62	46
44	What is the optimum timing of hernia repair after successful reduction in cases of acute incarceration?	60	48
2	What is the incidence of post-traumatic stress disorder (PTSD) following emergency surgery?	56	44
28	How does patient fatigue affect surgical outcomes after emergency surgery?	53	49
8	Do outcomes after paediatric appendicectomy vary in a district general hospital setting when compared with a tertiary hospital setting?	50	64
36	Is there any benefit to taking a swab of pus when performing incision and drainage of an abscess?	49	74
42	Is laparoscopic approach feasible in emergency inguinal hernia?	47	73
38	What are the indications for low-pressure peritoneum in emergency abdominal surgery and do we have data on outcomes?	41	69
12	Is there a difference in clinical outcomes between methods currently employed to secure the appendix stump during laparoscopic appendicectomy?	36	74
40	Is there a role for robotic-assisted surgery in adult patients with emergency surgical conditions?	24	111
*Emergency UGI surgery*			
2	Does a ‘hot gallbladder’ pathway reduce sepsis rates in addition to gallstone-related complications?	100	18
7	Which patient cohort derives the greatest clinical benefit from index cholecystectomy for acute cholecystitis?	100	20
12	Can mild acute pancreatitis be managed in an ambulatory setting and in which patients is it safe to do so?	95	31
5	Should every centre that offers emergency general surgery offer a ‘hot gallbladder’ service? Which model is best and is pre-operative magnetic resonance cholangiopancreatography (MRCP) required?	93	21
4	Should management of acute cholecystitis in the geriatric population differ from standard care?	88	19
8	Is cholecystostomy adequate in cases of empyema gall bladder in an older adult, unfit patient or is interval cholecystectomy required?	87	26
11	Is therapeutic anticoagulation indicated in cases of portal vein thrombus secondary to acute severe pancreatitis?	85	25
6	Can the management of acute cholecystitis be safely performed in an ambulatory setting?	82	31
9	What is the best immediate severity assessment for acute pancreatitis (i.e. not a score that uses a 48-h window)?	77	40
3	Should intra-operative cholangiogram with or without stone retrieval (by common bile duct exploration if necessary) be routine in cholecystectomy for acute cholecystitis?	60	49
10	What is the optimal management of alcohol-related pancreatitis?	55	52
*Emergency colorectal surgery*			
2	Does a formal cancer resection improve disease-free and overall survival in perforated or obstructing emergency colorectal cancer resections?	102	15
6	Is endoscopic follow-up required in CT-confirmed acute diverticulitis?	93	25
1	How do stent, stoma or immediate resection for obstructing colorectal cancers compare?	92	15
14	Regarding the various treatment strategies in lower gastrointestinal bleeding (e.g. surgical, endoscopy, interventional radiology, medical systemic treatment), is single or combination treatments superior?	81	30
9	Is there a CRP level (or other marker) which could identify cases of diverticulitis which can safely be managed conservatively, i.e. antibiotics versus no antibiotics?	80	33
12	What is the incidence and indications/selection criteria for surgical intervention with sigmoid volvulus?	80	20
8	Could FiT testing be used to screen for possible colorectal malignancy in diverticulitis patients rather than routine post-admission endoscopy/CT Colonography?	79	30
5	What are oncologic results in colon cancer treated in an emergency situation?	77	34
15	What is the optimum management of novel oral anticoagulants (NOACs) in patients with PR bleeding?	75	28
4	Endoscopic obstructing colorectal tumour: what should we do and what is the best timing for surgical intervention?	67	23
16	Should we train surgeons to mark for stomas in the emergency setting and would this affect patient satisfaction with stoma?	67	37
10	Should perforated diverticular disease be operated on exclusively by colorectal surgeons to decrease the rate of Hartmann’s procedure?	61	48
17	What is the evidence comparing diverting stoma and the use of Flexi-Seal in necrotising perineal and perianal infections?	60	42
11	Is there a role and benefit of laparoscopic washout for mild diverticulitis and how does it correlate with the Hinchey scale?	54	51
3	What is the best operative strategy to achieve laparoscopic resection in obstructing left sided colorectal cancers?	53	39
13	Is there a role for, and what would the indications be, for a laparoscopic Hartmann’s procedure for sigmoid volvulus in older adults?	46	52
7	Has the age at time of presentation of acute diverticulitis changed in the last 20 years and why?	35	57
*Non-technical questions*			
1	Which human/organisational factors cause inefficiency in a dedicated emergency theatre?	103	14
2	What influences decision-making for emergency surgery for the surgeon, patient and family?	93	13
3	How can the informed consent process be optimised for emergency general surgery patients?	84	17
4	What are the costs and ethical considerations of randomisation in emergency surgery?	63	40
*Health service related*			
12	Are emergency surgical outcomes improved with peri-operative physician or geriatrician input?	96	15
1	Which patients derive the greatest benefit from routine HDU/ITU care after emergency surgery?	92	15
10	Is there a volume–outcome relationship for emergency surgery?	86	17
13	Are there improved outcomes in patients who are treated on a triaged speciality take? (e.g. pancreatitis to UGI, colon cancer to colorectal)	85	22
11	What effect does a fast-track service have on the overall EGS service for patients requiring urgent cholecystectomy, appendicectomy and hernia surgery?	84	17
14	Is there a need for an ‘Emergency Surgery’ sub-specialisation?	82	24
2	Is there a benefit when a senior decision-maker sees the patient first?	79	32
3	Are rapid acute surgical assessment units cost-effective?	74	29
8	Would direct access to US and CT imaging for general practitioners affect triage of emergency referrals in NHS?	66	35
9	Should surgical emergencies be managed by a specialist or a generalist team?	65	35
5	What are the optimum and acceptable extended criteria for referral to surgical ambulatory care for emergency referrals?	64	37
6	What is the level of collaboration between dedicated emergency general surgeons and sub-specialists, hepatobiliary (HPB), upper gastrointestinal (UGI) and lower gastrointestinal?	59	46
4	What are the reasons for the increase in emergency referrals to secondary care in the UK?	56	46
7	Do emergency general surgery consultant surgeons require an elective component within their job plan and should they develop an elective subspecialty?	55	39

**Table 2:** Final list of questions following prioritisation in Phase 2

*General EGS*	
Which pre- or post-operative interventions improve clinical outcomes in patients with frailty after emergency surgery?	
How can training be improved to meet emergency general surgery demand, including open surgery?	
What is the impact of emergency laparotomy on quality of life in frail patients?	
What are the best predictors of adverse outcomes and mortality in older adults presenting with emergency general surgery pathology?	
How does small bites closure compare to traditional mass closure in the emergency setting (wound infection, fascial dehiscence and incisional hernia rates)?	
*Emergency colorectal surgery*	
Does a formal cancer resection improve disease-free and overall survival in perforated or obstructing emergency colorectal cancer resections?	
How do stent, stoma or immediate resection for obstructing colorectal cancers compare?	
What are the absolute risks of anastomotic leak following emergency small bowel/large bowel anastomosis, and the relative importance of risk factors?	
How can we optimise the rate of reversal of Hartmann’s when performed as an emergency and what are the predictive factors in those who are subsequently reversed?	
Does a laparoscopic approach for adhesional small bowel obstruction reduce the risk of recurrence, and how do we identify those who would benefit most from laparoscopic approach?	
*Non-technical*	
Which human/organisational factors cause inefficiency in a dedicated emergency theatre?	
*Health service related*	
Are emergency surgical outcomes improved with perioperative physician or geriatrician input?	
Is there a need for an ‘Emergency Surgery’ sub-specialisation?	
Is there a volume–outcome relationship for emergency surgery?	
What is the value of enhanced recovery after surgery (ERAS) programmes for EGS patients, compared to standard management?	
Which patients derive the greatest benefit from routine high-dependency unit (HDU)/intensive treatment unit (ITU) care after emergency surgery?	
Should patients have extended venous thromboembolism (VTE) prophylaxis after an emergency laparotomy with or without resection?

**Table 3:** Questions put forward for Phase II prioritisation

Question	High-priority ranking (4 or 5) (*n*)	Low-priority ranking (1 or 2) (*n*)
*General EGS*		
Which pre- or post-operative interventions improve clinical outcomes in patients with frailty after emergency surgery?	130	13
How can training be improved to meet emergency general surgery demand, including open surgery?	126	10
What is the impact of emergency laparotomy on quality of life in frail patients?	123	14
What are the best predictors of adverse outcomes and mortality in older adults presenting with emergency general surgery pathology?	120	21
What are the absolute risks of anastomotic leak following emergency small bowel/large bowel anastomosis, and the relative importance of risk factors?	112	10
How can we optimise the rate of reversal of Hartmann’s when performed as an emergency and what are the predictive factors in those who are subsequently reversed?	111	21
Does a laparoscopic approach for adhesional small bowel obstruction reduce the risk of recurrence, and how do we identify those who would benefit most from laparoscopic approach?	111	18
What is the value of enhanced recovery after surgery (ERAS) programmes for EGS patients, compared to standard management?	108	17
How does small bites closure compare to traditional mass closure in the emergency setting (wound infection, fascial dehiscence and incisional hernia rates)?	108	18
Should patients have extended venous thromboembolism (VTE) prophylaxis after an emergency laparotomy with or without resection?	105	28
What is the optimal method of assessing fitness for surgery in the emergency setting?	104	14
How does negative pressure wound therapy affect clinical outcomes after emergency surgery?	103	24
Are outcomes after emergency surgery improved with the laparoscopic approach as compared to the open approach?	96	25
Is the use of mesh safe for the repair of a strangulated hernia where there has been small bowel contamination via enterotomy?	89	35
Which cohort of EGS patients derive the greatest benefit from post-operative chest physio?	81	33
What is the optimum timing of hernia repair after successful reduction in cases of acute incarceration?	71	44
*Emergency UGI surgery*		
Should management of acute cholecystitis in the geriatric population differ from standard care?	106	23
Does a ‘hot gallbladder’ pathway reduce sepsis rates in addition to gallstone-related complications?	104	20
Should every centre that offers emergency general surgery offer a ‘hot gallbladder’ service? Which model is best and is pre-operative MRCP required?	104	263
Is therapeutic anticoagulation indicated in cases of portal vein thrombus secondary to acute severe pancreatitis?	98	27
Can mild acute pancreatitis be managed in an ambulatory setting and in which patients is it safe to do so?	98	31
Is cholecystostomy adequate in cases of empyema gall bladder in an older adult unfit patient or is interval cholecystectomy required?	96	32
*Emergency colorectal surgery*		
Does a formal cancer resection improve disease-free and overall survival in perforated or obstructing emergency colorectal cancer resections?	120	19
How do stent, stoma or immediate resection for obstructing colorectal cancers compare?	119	10
Endoscopic obstructing colorectal tumour: what should we do and what is the best timing for surgical intervention?	105	21
What are oncologic results in colon cancer treated in an emergency situation?	102	28
Is endoscopic follow-up required in CT-confirmed acute diverticulitis?	95	27
Regarding the various treatment strategies in lower gastrointestinal bleeding (e.g. surgical, endoscopy, interventional radiology, medical systemic treatment), is single or combination treatments superior?	94	35
Could FIT testing be used to screen for possible colorectal malignancy in diverticulitis patients rather than routine post-admission endoscopy/CT Colonography?	85	33
Is there a CRP level (or other marker) which could identify cases of diverticulitis which can safely be managed conservatively, i.e. antibiotics versus no antibiotics?	83	39
What is the best operative strategy to achieve laparoscopic resection in obstructing left-sided colorectal cancers?	79	47
What is the incidence and indications/selection criteria for surgical intervention with sigmoid volvulus?	75	28
*Non-technical*		
Which human/organisational factors cause inefficiency in a dedicated emergency theatre?	119	14
What influences decision-making for emergency surgery for the surgeon, patient and family?	119	23
*Health service related*		
Are emergency surgical outcomes improved with peri-operative physician or geriatrician input?	117	22
Is there a need for an ‘Emergency Surgery’ sub-specialisation?	115	24
Is there a volume–outcome relationship for emergency surgery?	112	20
What effect does a fast-track service have on the overall EGS service for patients requiring urgent cholecystectomy, appendicectomy and hernia surgery?	105	23
How can the informed consent process be optimised for emergency general surgery patients?	95	32
Is there a benefit when a senior decision-maker sees the patient first?	87	28
Are there improved outcomes in patients who are treated on a triaged speciality take? (e.g. pancreatitis to UGI, colon cancer to colorectal)	87	23

## Abbreviations

EGS: Emergency General Surgery
NHS: National Health Service
UK: United Kingdom
NELA: National Emergency Laparotomy Audit
SSRG: Scottish Surgical Research Group
WSES: World Society of Emergency Surgery
ASGBI: Association of Surgeons of Great Britain and Ireland
GDPR: General Data Protection Regulation
ERAS: Enhanced Recovery after Surgery
HDU: High-dependency unit
ITU: Intensive treatment unit
VTE: Venous thromboembolism
NECOPD: National Confidential Enquiry into Patient Outcomes and Deaths
NELA: National Emergency Laparotomy Audit
EPOCH: Enhanced Peri-operative Care for High-Risk Patients
ELC: Emergency Laparotomy Collaborative
ELLSA: Emergency Laparoscopic and Laparotomy Scottish Audit
ELF: Emergency Laparotomy and Frailty
CT: Computed tomography
POCUS: Point-of-care ultrasound scan
PTSD: Post-traumatic stress disorder
MRCP: Magnetic resonance cholangiopancreatography
CRP: C-reactive protein (need to write this in words)
FIT: Faecal immunochemical testing (need to write this)
NOACs: Novel oral anticoagulants
PR: Per rectal
US: Ultrasound
HPB: Hepatobiliary
UGI: Upper gastrointestinal
